# Extracorporeal photopheresis alone or in combination with ruxolitinib for the treatment of chronic graft-versus-host disease

**DOI:** 10.3389/fimmu.2026.1802710

**Published:** 2026-04-22

**Authors:** Christian Koch, Markus G. Manz, Mirjam Nägeli, Dominik Schneidawind

**Affiliations:** 1 Department of Medical Oncology and Hematology, University Hospital Zurich, Zurich, Switzerland; 2 Department of Dermatology, University Hospital Zurich, Zurich, Switzerland; 3 Comprehensive Cancer Center Zurich, University Hospital Zurich and University of Zurich, Zurich, Switzerland; 4 Department of Medicine II, Faculty of Medicine, University of Tübingen, Tübingen, Germany

**Keywords:** allogeneic hematopoietic stem cell transplantation, chronic graft-versus-host disease, extracorporeal photopheresis (ECP), immunomodulation, JAK-STAT signaling pathway, ruxolitinib

## Abstract

Chronic graft-versus-host disease (cGvHD) is a major cause of morbidity after allogeneic hematopoietic stem cell transplantation. Ruxolitinib (RUX) is a standard second-line treatment for steroid-refractory or -dependent cGvHD, while extracorporeal photopheresis (ECP), an autologous cell-based immunomodulatory procedure, is also widely used. However, comparative real-world data on combined immunomodulation with the RUX-ECP combination are scarce. We conducted a retrospective single-center analysis of patients with steroid-refractory or -dependent cGvHD receiving RUX-ECP (n=30) or ECP alone (n=21) between 2012 and 2025. The overall response rate was 77% with RUX-ECP and 52% with ECP (p=0.13), with CR rates of 17% and 10% (p=0.69). RUX-ECP was associated with a significantly shorter time to first response (2.6 vs. 12.3 months, p=0.0249). Organ-specific trends favored RUX-ECP in gastrointestinal, ocular and cutaneous cGvHD, whereas both regimens showed limited activity in pulmonary disease. Overall survival, relapse incidence and non-relapse mortality were comparable. At 12 months, complete steroid discontinuation (69% vs. 10%; p=0.005) and relative corticosteroid reduction were significantly greater with RUX-ECP (88% vs. 30%; p=0.0026). Toxicities of RUX-ECP were manageable and consistent with the known RUX profile, and several patients discontinued therapy after stable responses. Thus, combined immunomodulation with RUX-ECP showed high and fast response rates, a favorable safety profile and substantial steroid-sparing, supporting further evaluation in steroid-refractory or -dependent cGvHD patients.

## Introduction

Chronic graft-versus-host disease (cGvHD) is the most frequent late complication after allogeneic hematopoietic stem cell transplantation (HSCT) and a major cause of morbidity, impaired quality of life, and non-relapse mortality (1). It results from dysregulated interactions among donor-derived immune cells and aberrant cytokine signaling, driving inflammation, tissue injury and fibrosis that lead to the clinical manifestations of cGvHD (2–5).

Despite improved donor selection and immunosuppression, cGvHD still affects up to 50% of patients. Corticosteroids remain the first-line therapy, but many patients develop steroid-refractory or -dependent disease. The JAK1/2 inhibitor ruxolitinib (RUX) has become the standard second-line therapy (6).

Extracorporeal photopheresis (ECP) is an autologous, cell-based immunomodulatory therapy with established efficacy in cGvHD and a favorable safety profile (7–10). In ECP, peripheral leukocytes are collected, treated ex vivo with 8-methoxypsoralen and UVA, and reinfused. The resulting apoptotic cells are thought to induce immune tolerance via antigen-presenting cell reprogramming and expansion of regulatory pathways, which may complement JAK inhibitor-mediated cytokine blockade. ECP is also widely used in other immune-mediated settings, including solid organ transplant rejection.

Importantly, the 2024 EBMT guidelines emphasize that the optimal integration of ECP in the ruxolitinib era remains unclear and highlight combination strategies - including ECP as a partner therapy - as a key research priority (11). Direct comparative data on RUX and ECP, particularly in combination, are scarce, and whether combined use provides additive benefit is still uncertain (12, 13).

Here, we report real-world outcomes from a retrospective single-center cohort of patients with cGvHD treated with ECP alone or RUX-ECP, evaluating organ-specific and overall responses, response kinetics, survival outcomes, immunosuppression tapering, and safety.

## Materials and methods

### Patients

Patients diagnosed with cGvHD and treated with RUX-ECP or ECP at the University Hospital Zurich between January 2012 and September 2025 were retrospectively identified through institutional database searches. Eligibility required steroid-refractory or -dependent cGvHD according to NIH consensus definitions. Patients were included only if RUX-ECP or ECP was administered continuously without major treatment interruptions. During the study period, 53 patients with cGvHD received RUX-ECP or ECP at our institution, of whom 51 (96%) were included in the study cohort. Two patients were excluded due to very short ECP exposure (2 and 3 cycles, respectively), considered insufficient for response assessment. The study was conducted in accordance with the Declaration of Helsinki and approved by the local ethics committee (BASEC number 2025-01520). Treatment allocation was not randomized and reflected evolving clinical practice. ECP monotherapy was predominantly used before ruxolitinib became routinely available/reimbursed, whereas after availability, combined RUX-ECP was preferentially used.

### Diagnosis and grading of cGvHD

Diagnosis, overall severity and organ involvement of cGvHD were established according to NIH consensus criteria (14) with organ manifestations graded on a scale from 0-3. Mild disease was defined as involvement of one to two organs with grade 1 manifestations; moderate disease as involvement of three or more organs with grade 1 manifestations or at least one organ with grade 2, or lung involvement with grade 1; and severe disease as at least one organ with grade 3 or lung involvement with grade 2 or 3.

### Treatment with RUX-ECP or ECP

ECP was administered on two consecutive days every 2–4 weeks per institutional standards. The photopheresis treatment was performed using the THERAKOS® system and heparin as an anticoagulant. Patients allocated to the ECP group received ECP as their sole treatment throughout the observation period. A smaller subset of patients (n=7) initially received ECP for a sufficient treatment duration (≥ 6 months) and subsequently transitioned to combined therapy. In these cases, follow-up under ECP was censored at the time of RUX initiation, and all study parameters were reassessed from that point onward within the RUX-ECP cohort, so that each treatment period was analyzed independently. RUX-ECP was defined as concurrent administration of ruxolitinib and ECP, independent of which therapy was initiated first. Patients in the RUX-ECP group received RUX orally twice daily at 5–10 mg, while ECP was administered as two consecutive sessions every 2–4 weeks. When the two treatments were started at different times, only the overlap period during which both therapies were administered concurrently was included for response and time-to-response analyses, with the index date defined as initiation of the second therapy. Dose and interval adjustments (RUX dose modifications; ECP intervals of 2–4 weeks) were performed at physician discretion based on toxicity and clinical course, reflecting real-world practice.

### Response assessment

Organ-specific response was assessed retrospectively according to NIH consensus criteria (15), based on contemporaneous clinical documentation and objective parameters, including laboratory values and standardized functional assessments. Overall response was defined as follows: complete response (CR), resolution of all cGvHD-related manifestations; partial response (PR), improvement of at least one affected organ by ≥1 NIH grade without progression in any other organ; stable disease (SD), no improvement in any affected organ; progressive disease (PD), defined as SD with additional worsening of at least one organ system. Time to first response was defined as the interval from treatment initiation to the first documented CR or PR. For pulmonary cGvHD, forced expiratory volume in 1 second (FEV1) and for sclerotic skin GvHD, the modified Rodnan skin score (mRSS) was serially assessed by a dermatologist. Reduction of concomitant immunosuppression (corticosteroids and cyclosporine A (CsA)) was evaluated as a separate endpoint and was not incorporated into the overall response definition.

### Data collection and safety

Clinical data were retrospectively collected from electronic patient records covering the period from the start of RUX-ECP or ECP. Adverse events (AEs) were graded according to the Common Terminology Criteria for Adverse Events (CTCAE), version 5.0 (16). In clinical practice, grade 3–4 cytopenias typically prompted dose reduction to 5 mg twice daily or discontinuation of ruxolitinib. CMV reactivation was defined as detection of ≥500 CMV DNA copies/ml in peripheral blood. Antiviral therapy was generally initiated at ≥5000 copies/ml or earlier if clinically indicated, depending on symptoms and treating physician’s judgement.

### Clinical endpoints

Overall survival (OS) was defined as the time from initiation of ECP or RUX-ECP therapy to death from any cause, with surviving patients censored at last follow-up. Non-relapse mortality (NRM) was defined as death without prior relapse of the underlying malignancy. Relapse incidence (RI) was defined as recurrence or progression of the underlying disease after transplantation. Event-free survival (EFS) was defined as the time to relapse or non-relapse mortality, whichever occurred first.

### Statistical analysis

Statistical significance testing was performed using GraphPad Prism 10 (GraphPad Software Inc., San Diego, CA, USA). Continuous variables were compared using the Mann–Whitney U test; paired or longitudinal data were analyzed using the Wilcoxon matched-pairs test or repeated-measures one-way ANOVA. Categorical variables were compared with Fisher’s exact test, and time-to-event outcomes with the Kaplan-Meier method and log-rank test. A two-sided p-value < 0.05 was considered statistically significant.

## Results

### Patient characteristics

30 patients received RUX-ECP while 21 patients received ECP. Patient characteristics are shown in **Table 1**. All patients had previously received systemic corticosteroids. The proportion of male patients was 57% in the RUX-ECP group and 48% in the ECP group. The median age was 57 years (range 18-76) in the RUX-ECP group and 50 years (range 27-61) in the ECP group (p=0.047).

**Table 1:** Patient characteristics of the study cohort. Patient characteristics including age, sex distribution, underlying hematologic disease, donor type, conditioning regimen, and GvHD prophylaxis (p-values were calculated using Fisher’s exact test for categorical variables and Mann-Whitney test for continuous variables).

Characteristic	RUX-ECP	ECP	P-value
Baseline characteristics			
N	30	21	
% female	43	52	0.58
Median age at time of allogeneic HSCT (range)	54 (17-73)	48 (25-59)	0.12
Median age at time of start RUX-ECP or ECP (range)	57 (18-76)	50 (27-61)	0.047
Median BMI at start of RUX-ECP or ECP (range)	24 (17-38)	25 (16-33)	0.64
Underlying disease			
Acute myeloid leukemia (%)	12 (40)	8 (38)	> 0.99
Myelodysplastic neoplasms (%)	9 (30)	5 (24)	0.75
Myeloproliferative neoplasms (%)	2 (7)	1 (5)	> 0.99
MDS/MPN (%)	2 (7)	1 (5)	> 0.99
Acute lymphoblastic leukemia (%)	3 (10)	2 (10)	> 0.99
B-cell lymphomas (%)	0 (0)	1 (5)	0.41
T-cell lymphomas (%)	2 (7)	1 (5)	> 0.99
Multiple myeloma	0 (0)	2 (10)	0.16
Donor type			
Matched sibling donor (%)	11 (37)	10 (48)	0.57
Matched unrelated donor (%)	14 (47)	9 (43)	> 0.99
Haploidentical donor (%)	5 (17)	2 (10)	0.69
Conditioning intensity			
Reduced intensity conditioning (%)	18 (60)	11 (52)	0.78
Myeloablative conditioning (%)	12 (40)	10 (48)	0.78
GVHD prophylaxis			
Calcineurin inhibitor + MMF + ATG (%)	16 (53)	10 (48)	0.78
Calcineurin inhibitor + MTX + ATG (%)	6 (20)	3 (14)	0.72
Calcineurin inhibitor + MMF + ptCy (%)	5 (17)	1 (5)	0.38
Calcineurin inhibitor + MMF/MTX (%)	3 (10)	7 (33)	0.07
CMV status			
R+/D+ (%)	8 (27)	6 (29)	> 0.99
R-/D+ (%)	1 (3)	1 (5)	> 0.99
R+/D- (%)	8 (27)	4 (19)	0.53
R-/D- (%)	12 (40)	10 (48)	0.78
Not available	1 (3)	0 (0)	

Median follow-up was 31 months (range 3-100 months) for RUX-ECP and 80 months (range 6-158 months) for ECP (p=0.051), reflecting the earlier implementation of ECP (03/2012) than RUX-ECP (06/2017). Most patients had myeloid neoplasms, most frequently acute myeloid leukemia (AML; 40% RUX-ECP, 38% ECP) and myelodysplastic neoplasms (MDS; 30% RUX-ECP, 24% ECP). Most patients underwent matched sibling (MSD) or unrelated (MUD) donor transplantation. Reduced-intensity conditioning (RIC) was most commonly used (60% RUX-ECP, 52% ECP). For GvHD prophylaxis, the most frequent regimen was CsA plus mycophenolate mofetil (MMF) and antithymocyte globulin (ATG; 53% RUX-ECP, 48% ECP). ATG was given in 73% of RUX-ECP and 62% of ECP patients; post-transplant cyclophosphamide was given in 17% of RUX-ECP and 5% of ECP patients.

Characteristics of cGvHD in both cohorts are shown in **Supplementary Table 1**. Severe overall cGvHD was present in 26 patients (87%) in the RUX-ECP and 18 patients (86%) in the ECP group. The most frequently affected organ was the skin (60% RUX-ECP, 57% ECP), followed by gastrointestinal (57% vs. 38%) and ocular involvement (50% vs. 33%).

Median time from HSCT to treatment initiation was 2.3 years (range 0.3-19 years) in the RUX-ECP group and 2.1 years (range 0.7-11.5 years) in the ECP group. Time from cGvHD diagnosis to treatment initiation was shorter in the ECP group (median 19 months RUX-ECP, 8 months ECP; p=0.0432).

### Response to RUX-ECP and ECP

**Figure 1:**
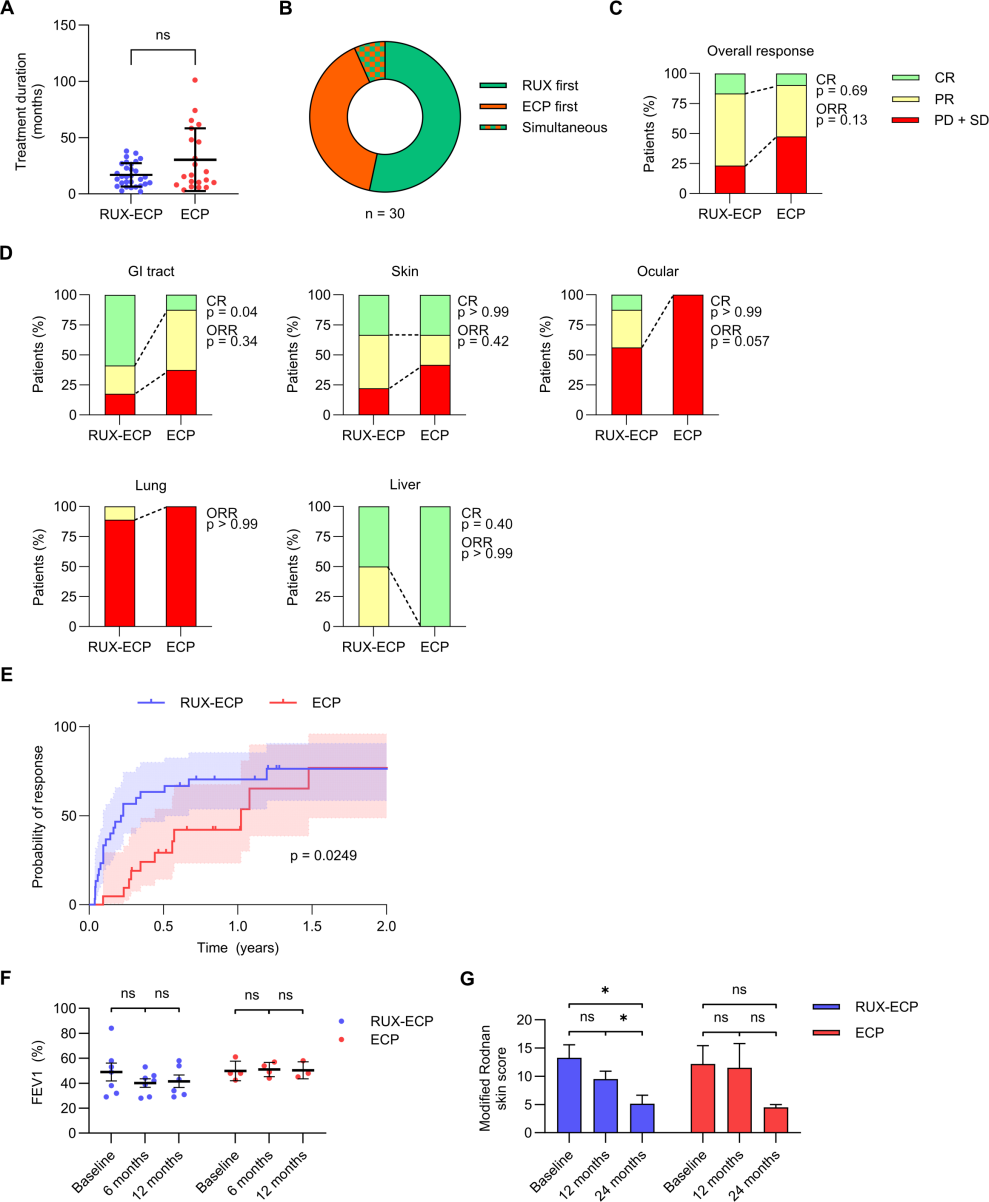
Treatment characteristics and response to RUX-ECP vs. ECP. **(A)** Treatment duration of RUX-ECP and ECP (Mann-Whitney-test; n.s., p>0.05). **(B)** Percentage of patients in the RUX-ECP group receiving RUX first, ECP first or simultaneous treatment. Patients that received both RUX and ECP within 14 days were regarded as “simultaneous”. **(C)** Overall response in the RUX-ECP and ECP groups, defined as the best documented treatment response during the treatment period. Complete response (CR) was defined as absence of cGvHD-related symptoms, partial response (PR) as ≥1 improvement in NIH score in at least one organ while no progression in another organ, stable disease (SD) as no change, and progressive disease (PD) as increase in NIH score (Fisher’s exact test). **(D)** Fractions of best organ-specific responses are depicted as stacked bars for RUX-ECP or ECP. Response during treatment was assessed using NIH consensus criteria (Fisher’s exact test). **(E)** Kaplan-Meier curve depicting time to first documented organ response for the RUX-ECP or ECP cohort (log-rank test). **(F)** Longitudinal FEV1 values in patients with pulmonary cGvHD under RUX-ECP or ECP are depicted (one-way ANOVA, n.s., p>0.05). **(G)** Longitudinal modified Rodnan skin scores were regularly assessed by a dermatologist in patients with sclerotic cutaneous cGvHD under RUX-ECP or ECP (one-way ANOVA, n.s., p>0.05; *p<0.05).

Treatment duration was comparable between RUX-ECP and ECP (median 15 months vs. 17 months, p=0.2554; **Figure 1A**). In the RUX-ECP group, 53% of patients received RUX prior to ECP while 40% started with ECP (**Figure 1B**). 7% of patients initiated RUX and ECP simultaneously, i.e., within 14 days.

At treatment initiation, concomitant immunosuppression was comparable between groups, with 66% of patients receiving steroids in both groups and 43% (RUX-ECP) vs. 48% (ECP) receiving CsA (p>0.99 and p=0.78). During treatment, complete response (CR) was achieved in 5 (17%) RUX-ECP and 2 (10%) ECP patients (p=0.69), while overall responses (CR + PR) were observed in 23 (77%) RUX-ECP and 11 (52%) ECP patients (p=0.13; **Figure 1C**).

Organ-specific response rates are summarized in **Figure 1D**. Gastrointestinal tract responses occurred in 82% of RUX-ECP patients vs. 63% in ECP (p=0.34), with significantly higher CR rates in the RUX-ECP group (59% vs. 13%, p=0.04). Skin responses were seen in 78% (RUX-ECP) vs. 58% (ECP; p=0.42), with CR rates of 33% in both groups (p>0.99). Across skin GvHD cases, 66% had sclerotic and 33% had non-sclerotic subtype, with no significant difference in responses between the two subtypes in both, the RUX-ECP and ECP groups (**Supplementary Table 2**). Ocular responses were achieved in 44% of RUX-ECP patients compared with none in the ECP group (p=0.057), with CR rates of 13% vs. 0% (p>0.99). Pulmonary responses were very limited (12.5% RUX-ECP vs. 0% ECP, p>0.99), with only one PR in the RUX-ECP group and no CR. Liver involvement showed 100% responses in both groups (p>0.99) and high CR rates (50% RUX-ECP and 100% ECP; p=0.40) with the majority of hepatic cGvHD cases harboring a cholestatic pattern (**Supplementary Table 3**).

The median time to first organ response was significantly shorter for RUX-ECP (2.6 vs. 12.3 months in ECP; p=0.0249; **Figure 1E**). To further objectively assess organ-specific responses, pulmonary function (FEV1) and skin fibrosis (mRSS) were serially evaluated during treatment. No improvement in lung function was documented in either group (**Figure 1F**), while significant reduction in skin fibrosis as measured by mRSS was observed in the RUX-ECP group after 24 months (**Figure 1G**).

### Clinical outcomes

**Figure 2:**
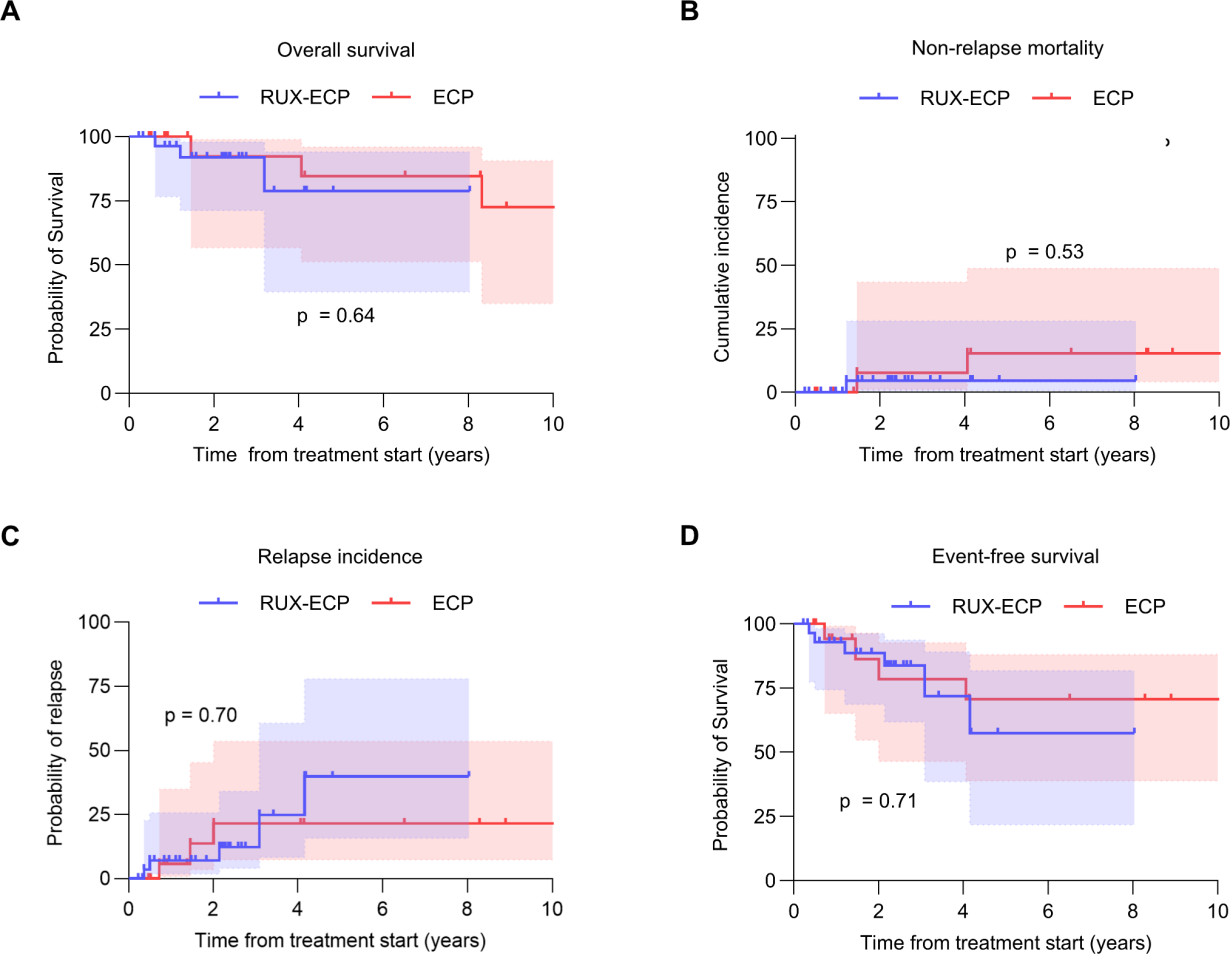
Survival outcomes and relapse incidence in patients treated with RUX-ECP versus ECP. **(A)** Kaplan-Meier survival curve depicting overall survival in the RUX-ECP and ECP cohorts (log-rank test). **(B)** Kaplan-Meier curve depicting non-relapse mortality in the RUX-ECP and ECP cohorts (log-rank test). **(C)** Kaplan-Meier curve depicting relapse incidence in the RUX-ECP and ECP cohorts (log-rank test). **(D)** Kaplan-Meier survival curve depicting event-free survival in the RUX-ECP and ECP cohorts (log-rank test).

OS was comparable in both groups, with median OS not reached during follow-up (p=0.64, **Figure 2A**). The 2-year OS was 92% for both groups. The 2-year NRM was 5% for RUX-ECP and 8% for ECP, respectively (p=0.53, **Figure 2B**). Relapse incidence was similar between groups (7% RUX-ECP vs. 14% ECP at 2 years, p=0.70; **Figure 2C**) as was event-free survival (89% RUX-ECP vs. 86% ECP at 2 years, p=0.71; **Figure 2D**).

### Reduction of immunosuppression and durability of response

**Figure 3:**
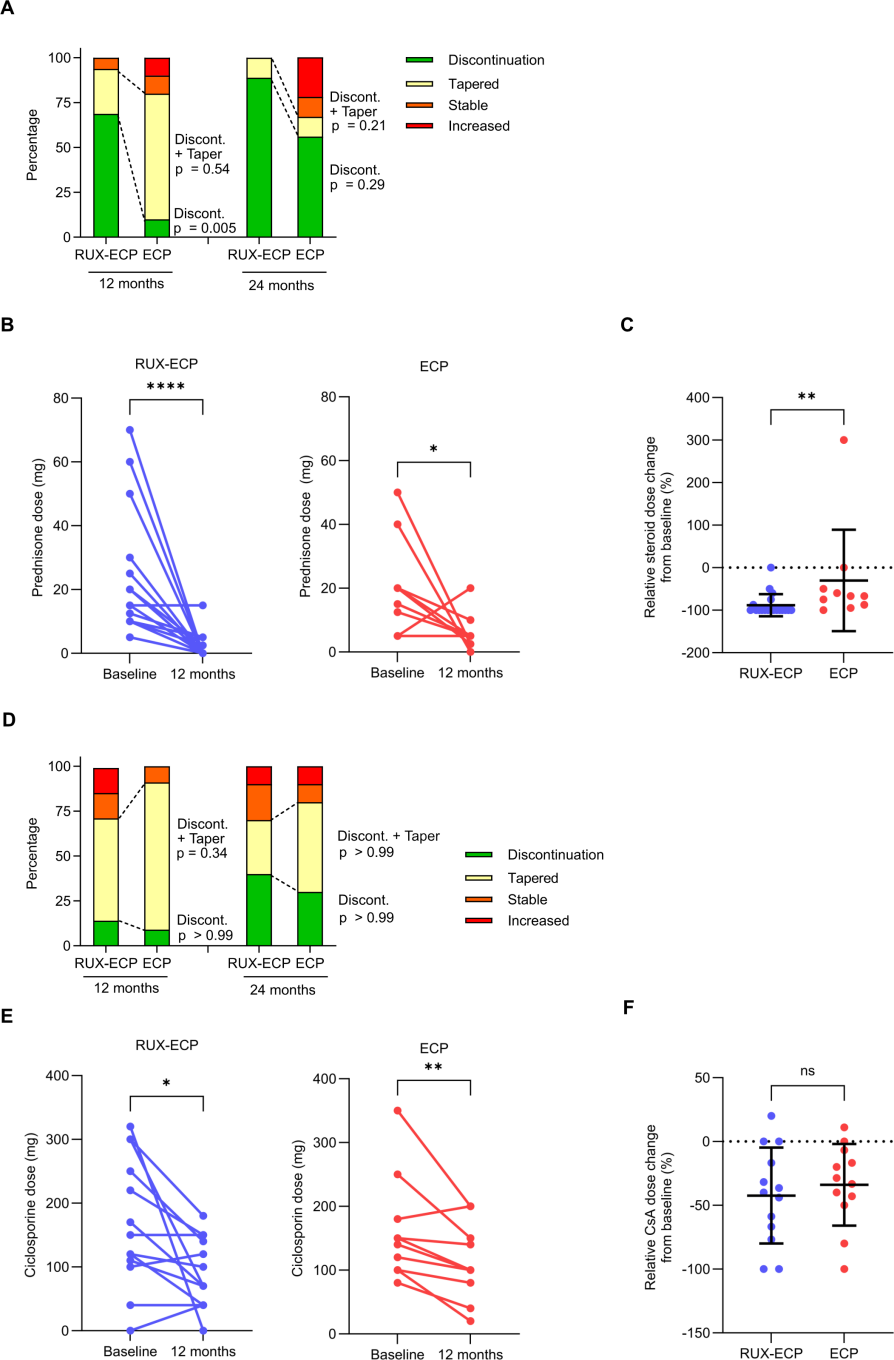
Reduction of immunosuppression under RUX-ECP or ECP. **(A)** Stacked bar graphs showing the proportion of patients who achieved complete steroid discontinuation, partial tapering, stable dose, or higher dose after 12 and 24 months of RUX-ECP or ECP. Upper p-values indicate differences in cumulative reduction, i.e. tapering and discontinuation, whereas lower p-values indicate differences in discontinuation rates between groups (Fisher’s exact test). **(B)** Absolute prednisone doses (mg per day) for each patient at time of treatment start and 12 months after RUX-ECP or ECP (Wilcoxon-test; *p < 0.05; ****p < 0.0001). **(C)** Relative steroid dose at 12 months compared to baseline between RUX-ECP and ECP (Mann-Whitney test; **p < 0.01). **(D)** Stacked bar graphs showing the proportion of patients who achieved complete CsA discontinuation, partial tapering, stable dose, or higher dose after 12 and 24 months of RUX-ECP or ECP. Upper p-values indicate differences in cumulative reduction and discontinuation rate, whereas lower p-values indicate differences in discontinuation rates between groups (Fisher’s exact test). **(E)** Absolute CsA doses (mg per day) for each patient at time of treatment start and 12 months after RUX-ECP or ECP (Wilcoxon-test; *p < 0.05; **p < 0.01). **(F)** Relative CsA dose at 12 months compared to baseline between RUX-ECP and ECP (Mann-Whitney test; n.s. p>0.05).

At 12 months, corticosteroid discontinuation was achieved in 69% of patients in the RUX-ECP group vs. 10% in the ECP group (p=0.005; **Figure 3A**). The cumulative rate of steroid discontinuation or reduction was 94% vs. 80% (p=0.54). At 24 months, discontinuation rates were 89% for RUX-ECP vs. 56% for ECP (p=0.29), with cumulative discontinuation or reduction rates of 100% vs. 67% (p=0.21). Mean absolute prednisone doses were significantly reduced after 12 months in both groups, from 22.9 mg to 1.7 mg in the RUX-ECP group (p<0.0001) and from 20.3 mg to 6.0 mg in the ECP group (p=0.039) (**Figure 3B**). Relative steroid reduction after 12 months was significantly greater in RUX-ECP vs. ECP (mean reduction 88% vs. 30%, p=0.0026; **Figure 3C**).

At 12 months, CsA discontinuation was achieved in 14% of the RUX-ECP group and 9% of the ECP group (p>0.99; **Figure 3D**). The cumulative rate of CsA discontinuation or reduction was 71% vs. 91%, respectively (p=0.34). At 24 months, discontinuation rates were 40% for RUX-ECP and 30% for ECP (p>0.99), with cumulative discontinuation or reduction rates of 70% and 80%, respectively (p>0.99). Mean CsA doses decreased significantly in both groups (169.2 mg to 90 mg in RUX-ECP, p=0.011; 162 mg to 113 mg in ECP, p=0.0098; **Figure 3E**), while relative CsA dose reduction was similar between groups at 12 months (42% vs. 34%; p=0.56; **Figure 3F**).

Among patients achieving a response (PR or CR), 95% in the RUX-ECP group maintained a response throughout the follow-up period, with 67% of patients either discontinuing RUX, ECP, both treatments or reducing their RUX dose (**Supplementary Figures 1A, B**). In the ECP group, 82% of responders (CR and PR) maintained a stable response, and 73% were able to discontinue ECP after a sufficiently long duration of treatment.

### Safety

Adverse events were compared between patients treated with RUX-ECP and ECP alone, focusing on hematologic toxicity and CMV reactivation (**Supplementary Table 4**). Grade 3/4 anemia occurred in 20% of RUX-ECP patients vs. none in ECP (p=0.036). Grade 3/4 thrombocytopenia was observed in 3% vs. 0% (p>0.99), and grade 3/4 neutropenia in 7% vs. 0% (p=0.51) of cases. Dose reductions for RUX-related cytopenias were required in 23% (7/30) of patients. CMV reactivation occurred in 13% (4/30) of RUX-ECP vs. 5% (1/21) of ECP patients (p=0.39). Median time from RUX initiation to CMV reactivation was 56 days. Two patients in the RUX-ECP group required treatment for CMV, but no grade 3/4 CMV reactivation or CMV disease was observed in either cohort.

## Discussion

In this retrospective single-center analysis, we compared outcomes of cGvHD patients treated with RUX-ECP vs. ECP alone, focusing on overall and organ-specific responses, immunosuppression reduction, and safety. Baseline characteristics were balanced, with similar donor types, conditioning regimens, and GvHD prophylaxes, although patients in the RUX-ECP cohort were slightly older. Median follow-up was longer in the ECP cohort due to its earlier clinical use, while RUX was approved later in Switzerland.

To the best of our knowledge, this study represents the largest cohort of cGvHD patients treated with RUX-ECP reported to date, providing real-world comparative evidence on a combination immunomodulatory strategy integrating a targeted small-molecule inhibitor with a cell-based therapy. RUX-ECP was associated with numerically higher overall and complete response rates compared with ECP alone (77% vs. 52% and 17% vs. 10%, respectively), although these differences did not reach statistical significance. Our findings are consistent with a previously published single-center report of 23 patients receiving RUX-ECP in cGvHD, which reported a comparable response rate of 74% with 9% CRs (13). The higher response and CR rate of RUX-ECP observed in our study may be attributed to the longer treatment duration (median 15 vs. 6 months), suggesting that sustained combination immune modulation may be necessary for optimal responses.

In comparison, in the pivotal REACH3 phase III trial, RUX monotherapy achieved an overall response rate of 49.7% at 24 weeks in a cohort with 58.8% severe cGvHD (6). More recent real-world series of RUX have reported higher response rates of up to 77%, though the proportion of patients with severe disease was lower (38%) than in our analysis (17). Notably, a recent acute GvHD study showed a reduced incidence of subsequent cGvHD with RUX-ECP compared to RUX alone (18), supporting the biologic plausibility of additive effects when combining JAK inhibition with ECP-mediated immune tolerance mechanisms.

Consistent with Maas-Bauer et al. (13), we observed highest response rates of RUX-ECP in gastrointestinal and skin cGvHD (82% and 78%, respectively). These rates were higher than previously reported, likely due to differences in treatment duration. While the median duration of RUX-ECP in Maas-Bauer et al. was 6 months (range 1-27 months), with some patients receiving as little as 1 month, our cohort had a median duration of 15 months, with only 7% receiving less than 6 months. This was particularly evident in sclerotic skin involvement, where early responses were modest but longitudinal mRSS assessments showed significant improvement with prolonged therapy, underscoring the importance of sustained treatment to achieve regression of skin fibrosis. Notably, ocular cGvHD showed relatively high response rates in our study (44%) compared with 20% reported by Maas-Bauer and colleagues.

Pulmonary cGvHD showed no objective improvement in either group. Although FEV1 remained stable in most cases, no measurable lung function recovery was observed, likely due to the irreversible nature of pulmonary cGvHD. However, stabilization suggests a clinically relevant effect (19), further highlighting the ongoing need for effective therapies.

A key finding of our study was the significantly shorter time to response with RUX-ECP compared to ECP (median 2.6 vs. 12.3 months). This difference had clinical implications: delayed responses under ECP were associated with prolonged corticosteroid exposure, with only 10% of patients off steroids at 12 months compared to 69% in the RUX-ECP group. In the REACH3 study, the median time to response was 29 days among responders only (20), while in our study, across both responders and non-responders, the median time was 2.6 months, which accounts for the longer time observed in our cohort.

Consistent with our findings, a recent Delphi study identified RUX-ECP as particularly effective in facilitating steroid tapering in cGvHD (21). The early benefits of RUX-ECP support its early use in patients with high disease burden or refractory cGvHD.

Clinical outcomes, including OS, NRM, RI, and EFS, were comparable between groups. As expected, RUX-ECP was associated with RUX-specific toxicities, notably grade 3/4 anemia, and showed a trend towards CMV reactivation (22). While largely manageable through dose reduction, no excess NRM was observed. Notably, a substantial proportion of patients were able to discontinue RUX and/or ECP after stabilization, suggesting that temporary combination therapy may achieve durable immune control without indefinite exposure.

In summary, RUX-ECP was associated with a significantly faster time to first response compared with ECP alone and with earlier and more frequent steroid discontinuation. Trends toward improved organ responses, particularly in gastrointestinal, ocular, and cutaneous cGvHD, further support the potential benefit of combined therapy. These findings support prospective evaluation of RUX-ECP and systematic integration of targeted JAK inhibition with cell-based immunotherapies in steroid-refractory or steroid-dependent cGvHD.

This study has several limitations. Its retrospective, single-center design is inherently subject to bias and the patient population was highly heterogeneous, with varying underlying hematologic malignancies, conditioning regimens, prior therapies, and clinical courses of cGvHD, which may have influenced outcomes. Some patients initially treated with ECP later received RUX-ECP, introducing potential crossover bias. In addition, the relatively small sample size limited statistical power, particularly for safety endpoints such as infection risk. Follow-up differed between cohorts due to the earlier adoption of ECP (2012) compared with the later introduction of RUX-ECP (2017), a period during which supportive care and cGvHD management evolved, which may have influenced outcome assessments. Era-dependent treatment allocation also limited interpretation of a ruxolitinib monotherapy comparator, as ruxolitinib-only patients generally had less severe disease, introducing potential bias in assessing the additive effect of ECP.

Despite these limitations, this study benefits from close and prolonged follow-up, the largest reported cohort of patients with cGvHD treated with RUX-ECP to date, and careful assessment of overall and organ-specific responses, immunosuppression reduction, standardized pulmonary function testing and dermatologic scoring.

## Data Availability

The raw data supporting the conclusions of this article will be made available by the authors, without undue reservation.
