# Supplementary material for: Extracorporeal photopheresis alone or in combination with ruxolitinib for the treatment of chronic graft-versus-host disease
Source: Front Immunol. 2026 Apr 22;17:1802710. doi: 10.3389/fimmu.2026.1802710 (PMC13143746; doi:10.3389/fimmu.2026.1802710)
Supplement: Supplementary file 1 [file SupplementaryFile1.pdf]

SUPPLEMENTARY INFORMATION

Supplementary Tables

Supplementary Table 1. Chronic GvHD characteristics.

Overall severity and organ involvement according to NIH consensus criteria of cGvHD prior to treatment with RUX-ECP or ECP (Fisher's exact test).

Supplementary Table 2. Cutaneous cGvHD subtypes and their response to RUX-ECP or ECP (Fisher's exact test).

Supplementary Table 3. Hepatic cGvHD subtypes and their response to RUX-ECP or ECP.

Supplementary Table 4. Adverse events after RUX-ECP or ECP.

Most common adverse events, including grade 3/4 cytopenias and CMV reactivation, following RUX-ECP or ECP (Fisher's exact test).

Supplementary Figures

Supplementary Figure 1. Treatment Continuation and Response Durability in cGVHD Patients.

(A) Distribution of patients at the end of follow-up in the RUX-ECP and ECP groups, based on their ongoing therapy.
(B) Proportion of patients in the RUX-ECP and ECP groups who maintained or lost response during follow-up.

Table S1

	RUX-ECP	ECP	P-value
cGVHD overall severity			
Mild	0 (0)	0 (0)	> 0.99
Moderate	4 (13)	3 (14)	> 0.99
Severe	26 (87)	18 (86)	> 0.99
Organ affection			
Skin (% of patients)	18 (60)	12 (57)	> 0.99
Grade 1	4	2	
Grade 2	9	5	
Grade 3	5	5	
Liver (% of patients)	2 (7)	3 (14)	0.63
Grade 1	1	0	
Grade 2	1	3	
Grade 3	0	0	
GIT (% of patients)	17 (57)	8 (38)	0.26
Grade 1	8	2	
Grade 2	6	6	
Grade 3	3	0	
Eye (% of patients)	15 (50)	7 (33)	0.27
Grade 1	3	2	
Grade 2	8	5	
Grade 3	4	0	
Lung (% of patients)	8 (27)	6 (29)	> 0.99
Grade 1	0	1	
Grade 2	4	4	
Grade 3	4	1	

Table S2

	Treatment	N	CR	PR	SD/PD	ORR	CR rate	P-Value
Sclerotic	RUX-ECP	12	3	7	2	83%	25%	CR-rate p=0.64<br>ORR-rate p=0.34
	ECP	8	3	2	3	62.5%	37.5%	
Non-sclerotic	RUX-ECP	6	3	1	2	67%	50%	CR-rate p=0.57<br>ORR-rate p 0.99
	ECP	4	1	1	2	50%	25%	

Table S3

	Laboratory pattern	Histology	Treatment	Response
Case #1	Mixed hepatitic-cholestatic	Cholestatic pattern	RUX-ECP	CR
Case #2	Cholestatic pattern	Cholestatic pattern	RUX-ECP	PR
Case #3	Cholestatic pattern	-	ECP	CR
Case #4	Mixed hepatitic-cholestatic	Cholestatic pattern	ECP	CR
Case #5	Mixed hepatitic-cholestatic	-	ECP	CR

Table S4

	**RUX-ECP**	**ECP**	**P-value**
N	30	21	
Grade 3/4 anemia (%)	6 (20)	0 (0)	0.036
Grade 3/4 thrombopenia (%)	1 (3)	0 (0)	> 0.99
Grade 3/4 neutropenia (%)	2 (7)	0 (0)	0.51
CMV reactivation (%)	4 (13)	1 (5)	0.39

Fig. S1

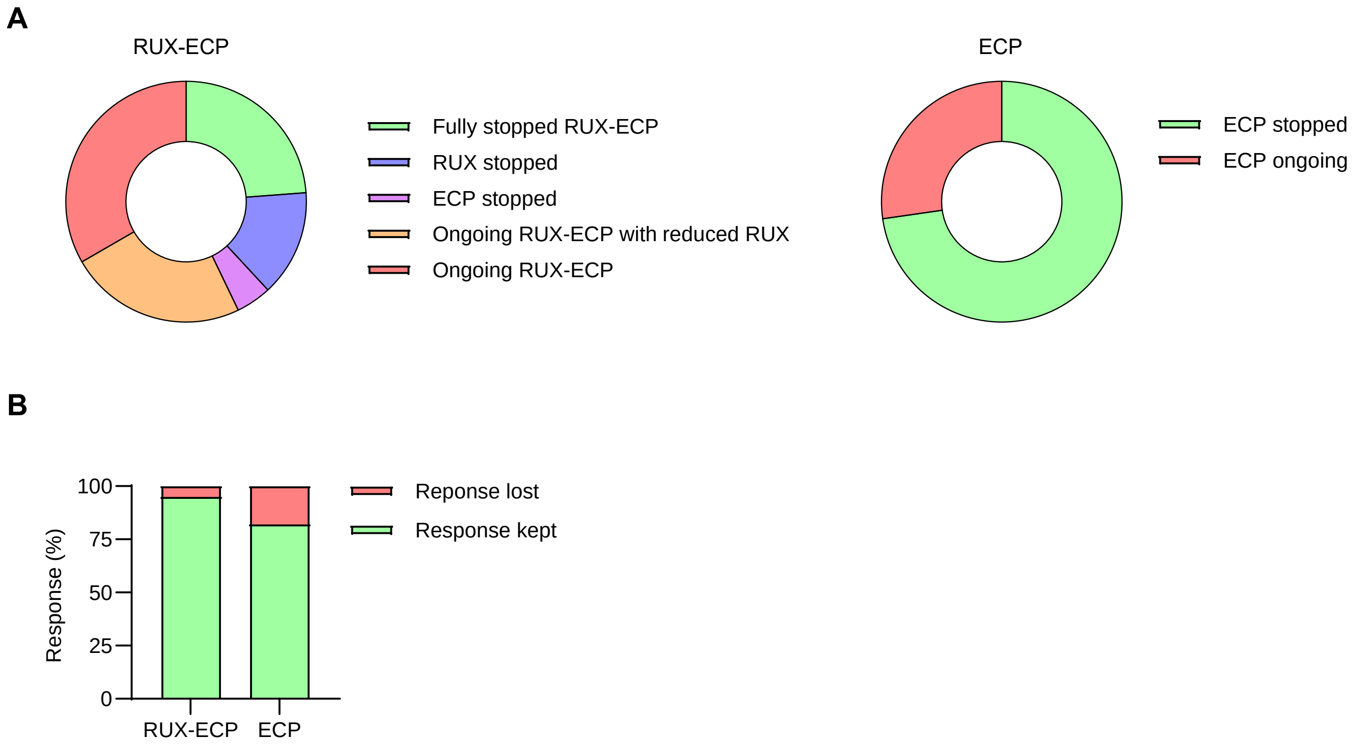
A
RUX-ECP
Fully stopped RUX-ECP
RUX stopped
ECP stopped
Ongoing RUX-ECP with reduced RUX
Ongoing RUX-ECP
ECP
ECP stopped
ECP ongoing
B
Response (%)
100
75
50
25
0
RUX-ECP
ECP
Reponse lost
Response kept
